# Safety and Efficacy of Minimum- or Zero-Contrast IVUS–Guided Percutaneous Coronary Interventions in Chronic Kidney Disease Patients: A Systematic Review

**DOI:** 10.3390/jcm10091996

**Published:** 2021-05-06

**Authors:** Alexandru Burlacu, Grigore Tinica, Crischentian Brinza, Radu Crisan-Dabija, Iolanda Valentina Popa, Adrian Covic

**Affiliations:** 1 Institute of Cardiovascular Diseases “Prof. Dr. George I.M. Georgescu”, 700503 Iasi, Romania; alexandru.burlacu@umfiasi.ro (A.B.); grigore.tinica@umfiasi.ro (G.T.); 2 Faculty of Medicine, University of Medicine and Pharmacy “Grigore T Popa”, 700115 Iasi, Romania; radu.dabija@umfiasi.ro (R.C.-D.); iolanda-valentina-g-popa@d.umfiasi.ro (I.V.P.); adrian.covic@umfiasi.ro (A.C.); 3 Medical Sciences Academy, 030167 Bucharest, Romania; 4 Pulmonology Department, Clinic of Pulmonary Diseases, 700115 Iasi, Romania; 5 Nephrology Clinic, Dialysis, and Renal Transplant Center, C.I. Parhon” University Hospital, 700503 Iasi, Romania

**Keywords:** chronic kidney disease, percutaneous coronary interventions, zero-contrast, minimum contrast, intravascular ultrasound, safety, efficacy

## Abstract

Conventional percutaneous coronary interventions (PCIs) frequently cause severe complications in chronic kidney disease (CKD) patients. Low-to-zero contrast intravascular ultrasound (IVUS) guided PCIs are promising alternatives in the CKD setting. We aim to systematically review up-to-date literature that have reported data and outcomes of low-to-zero contrast PCIs performed in CKD patients. We searched Embase, PubMed, and Cochrane databases for full-text articles that reported original data regarding efficacy and/or safety outcomes of IVUS-guided PCIs in patients with CKD. The quality of non-randomized trials included was assessed using the Newcastle–Ottawa scale. Six papers were included in the present systematic review: One non-randomized trial, two case series, and three case reports. Given the literature reported so far, contrast-free and IVUS-guided PCI procedures in patients with CKD appear to be safe (both in cardiac and renal outcomes) with a comparable efficacy to the conventional procedure, even in complex atherosclerotic lesions. No patient included in the mentioned studies showed renal function deterioration and did not need renal replacement therapy after the zero-contrast IVUS-guided percutaneous procedures. From a cardiovascular point of view, this technique proved to be safe in terms of cardiovascular outcomes. The undesirable consequences of conventional PCI in the CKD population might soon be effectively hampered by safer low-to-zero contrast IVUS-guided PCI procedures after a mandatory and rigorous evidence-based validation in long-awaited randomized controlled trials.

## 1. Introduction

Two of the most recent released clinical practice European Society of Cardiology Guidelines on coronary syndromes (e.g., acute from 2020 [1], and respectively, chronic from 2019 [2]) pointed to prominent and reliable recommendations (class I, level of evidence A) on minimizing the use of iodinated contrast agents during percutaneous coronary interventions (PCIs) in patients with severe chronic kidney disease (CKD) in order to prevent further deterioration and a subsequent increase in mortality [3,4]. Moreover, in this very setting, the same documents firmly suggested using low- or iso-osmolar contrast media at the lowest possible volume (class I indication, level of evidence A). 

Given the considerable number of worldwide patients receiving PCI and the significant proportion of patients with CKD, the issue of the amount of contrast used becomes troublesome, especially when the incidence of acute kidney injury (AKI) has risen from 5% up to 15% in the general population (elective versus acute settings) [5]. In addition, recent studies have reported that almost one out of three patients with severe CKD manifest AKI after PCI [6] with a significant rise in mortality on both short- and long-term [5,7]. Even a pre-procedural serum creatinine higher than 2.0 mg/dL proved to be an AKI indicator, strongly correlated with higher intra- and post-hospitalization mortality [5].

Based on the imperative of reducing contrast dose in PCI [8], a legitimate question arises: ‘How low can we go?’ [9], together with the concepts/techniques of ‘low-contrast’, ‘ultra-low contrast’ [9], ‘zero-contrast’ [10,11], and ‘no-contrast’ [12]. Briefly, the alternatives offered consist of performing PCI using digitally reconstructed roadmaps, intravascular ultrasound (IVUS), and fractional flow reserve (FFR) measured in real-time, given a previous angiography with minimal contrast (ultra-low angiography with 10 mL contrast) in another session [13].

Aiming to prevent further renal deterioration and the need for renal replacement therapy in severe CKD, this somewhat young strategy seems extremely attractive and has been explored in various studies. Moreover, two meta-analyses published last year (including 10, respectively 19 studies performed on the general population receiving IVUS-guided versus conventional PCIs) demonstrated substantial benefits of this approach in terms of safety and efficacy (including cardiovascular mortality) [14,15]. However, none of these two meta-analyses focused on low-contrast IVUS PCIs in advanced CKD patients since only few trials were aimed at this specific setting.

Our systematic review of papers examining low-contrast IVUS PCIs in a CKD setting is a first. Conducting this research is essential to assessing the current level of evidence and quality of publications and to highlighting the missing links before more extensive use in clinical practice. This is an important step towards safer interventional cardiovascular procedures in CKD and increased use of PCIs in CKD patients with coronary syndromes (as PCIs tend to be under-utilized in this high-risk setting [16]).

Our approach aims to systematically review up-to-date literature, gathering trials (either randomized or observational trials) that have reported data and outcomes (e.g., safety and efficacy) of minimum- to zero-contrast percutaneous coronary interventions explicitly performed in the setting of chronic renal disease patients with acute or chronic coronary syndromes.

## 2. Materials and Methods

The Preferred Reporting Items for Systematic Review and Meta-Analysis (PRISMA) checklist [17] was applied in each step of the systematic review conduction (Table S1).

### 2.1. Data Sources

We performed a literature search in Embase, PubMed, and Cochrane library databases from inception to 31 January 2021. The following search statement was used for the Embase database: “(‘percutaneous coronary intervention’/exp OR ‘percutaneous coronary intervention’) AND (‘chronic kidney disease’/exp OR ‘chronic kidney disease’ OR ‘CKD’ OR ‘nephropathy’ OR ‘kidney injury’ OR ‘kidney disease’) AND (‘low contrast’ OR ‘zero contrast’ OR ‘low-contrast’ OR ‘zero-contrast’)”. For Pubmed, the search was conducted using the following filters: “(‘percutaneous coronary intervention’ [MeSH Terms] OR ‘percutaneous coronary intervention’) AND (‘chronic kidney disease’ [MeSH Terms] OR ‘chronic kidney disease’ OR ‘CKD’ OR ‘nephropathy’ OR ‘kidney injury’ OR ‘kidney disease’) AND (‘low contrast’ OR ‘zero contrast’ OR ‘low-contrast’ OR ‘zero-contrast’)”. The Cochrane database was searched using the following statement: “‘percutaneous coronary intervention’ AND (‘chronic kidney disease’ OR ‘CKD’ OR ‘nephropathy’ OR ‘kidney injury’ OR ‘kidney disease’) AND (‘low contrast’ OR ‘zero contrast’ OR ‘low-contrast’ OR ‘zero-contrast’)”. Both MeSH and Emtree terms were used in the systematic search. The search was restricted to trials published in English. Two authors (A.B. and C.B.) independently screened titles and abstracts for the studies’ eligibility. Both authors identified separately the relevant articles fulfilling the inclusion criteria. Only those studies which were found eligible by both reviewers were included.

### 2.2. Study Selection

Trials were considered eligible if they fulfilled several criteria: Randomized or non-randomized studies, observational studies, case series, and case reports; adult humans aged > 18 years were included in the analysis; original data regarding efficacy and/or safety outcomes of IVUS-guided PCI in patients with CKD were reported; the definition of CKD was prespecified in concordance with international guidelines; a minimum-contrast or zero-contrast technique was performed; and IVUS-guided PCI technique was performed for acute or chronic coronary syndromes. In addition, several key exclusion criteria were set: Abstract-only studies; letters; unpublished data; meta-analyses; and inability to extract data. The inclusion criteria are summarized in Table 1 according to the PICOS statement.

### 2.3. Data Extraction

After a full-text examination of included studies, the following data were extracted: Study design; number of patients; patients’ age; clinical setting; CKD definition; type of intervention; comparator (when available); reported outcomes; follow-up duration; odds ratio (OR), risk ratio (RR), hazard ratio (HR), corresponding confidence interval (CI), *p*-value—when reported; and adverse renal and cardiovascular outcomes. Two authors (A.B. and C.B.) independently extracted the data. When there was a lack of consensus, the disagreement was resolved by a third senior reviewer (A.C.) and by consensus-based discussion.

### 2.4. Outcomes

We assessed cardiovascular efficacy outcomes of IVUS-guided PCI performed in patients with CKD and ACS or CCS reported in clinical studies, including at least one of the following: Cardiac death, all-cause death, stent thrombosis, revascularization, and myocardial infarction. In addition, we appraised safety outcomes reported in clinical trials, which included acute kidney injury after the procedure, requirement of renal replacement therapy (RRT), and worsening renal function.

### 2.5. Quality Assessment

The quality of non-randomized trials included in the present systematic review was appraised using the Newcastle–Ottawa scale, a tool composed of three domains: Selection of groups, comparability of groups, and evaluation of the outcomes of interest. Stars are assigned for each of the 8 essential items, with a maximum of 9 stars [18].

## 3. Results

**Figure 1:**
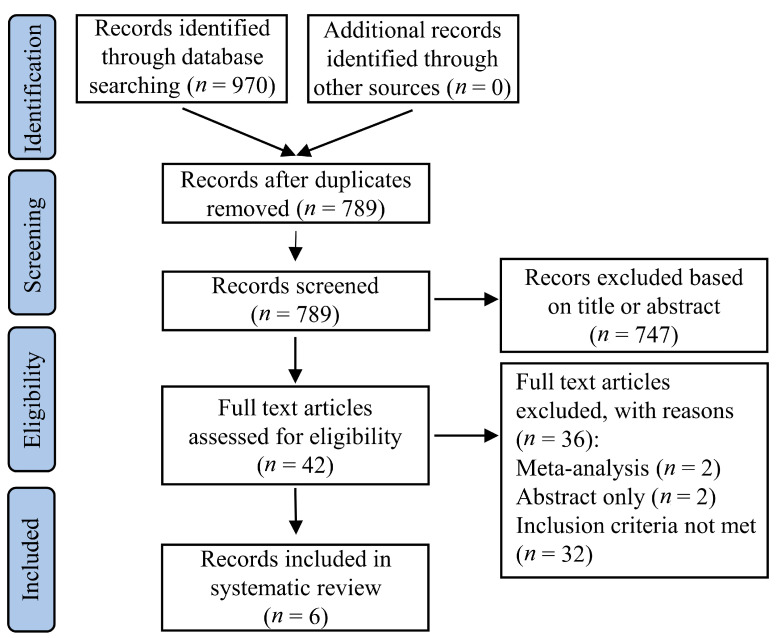
Flow diagram of the studies selection for inclusion in the systematic review.

Our search in prespecified databases retrieved 970 citations. After excluding 181 duplicates, 789 publications were left for screening. In addition, 749 citations were excluded after title or abstract screening, leaving 40 articles for full-text assessment. An additional 36 references were excluded after full-text reading, due to: Meta-analysis (2), abstract only (2), and inclusion criteria not being met (32), leaving six studies included in the systematic review (Figure 1).

General characteristics of the studies and the population included are summarized in Table 2.

Of the six papers included, five studies were single-center—USA (Ali, Patel, and Rahim) [12,13,20], Poland (Sacha) [10], India (Kumar) [19], while one study was performed in multiple centers from Japan (Sakai) [16]. Of the included studies, one trial was non-randomized (Sakai), two case series (Ali, Sacha), and three case reports (Kumar, Patel, and Rahim). Only one study compared minimum-contrast IVUS-guided PCI with angiography-guided PCI (Sakai). Regarding the PCI technique, two studies explored zero-contrast IVUS-guided PCI (Ali, Sacha), one study (Sakai) used minimum-contrast IVUS guided PCI, one study (Kumar) used IVUS-guided rota-assisted zero-contrast PCI, one study (Patel) used zero-contrast and minimum-contrast IVUS-guided PCI in the same procedure, and one study (Rahim) used zero-contrast PCI with a left ventricle support device. Outcomes of interest, as well as results reported in each study, are summarized in Table 3.

Sakai et al. included in their analysis only patients with advanced CKD (eGFR < 30 mL/min/1.73 m^2^), with the exception of patients with hemodialysis [16]. The authors included patients with three-vessel coronary disease and unprotected left main trunk disease in both groups, IVUS-guided PCI and angiography-guided PCI. Patients had previously underwent a classic angiography, then IVUS-guided PCI was performed, with minimum-contrast injection at the end of the procedure to exclude distal coronary complications. Less contrast volume was used in the case of patients treated with the IVUS-guided technique than in those with angiography-guided PCI (22 ± 20 mL versus 130 ± 105 mL, *p* = 0.001), with a similar PCI success rate between groups (99% versus 100%, *p* = 0.35).

Furthermore, patients from the IVUS group exhibited a significantly lower contrast-induced AKI rate than patients in the angiography group (2% versus 15%, *p* = 0.001). The rate of renal replacement therapy (RRT) at one year was lower in the minimum-contrast group (2.7% versus 13.6%, *p* = 0.01), though the composite endpoint of all-cause mortality, myocardial infarction, and RRT was similar among both groups (10.7% for IVUS-guided PCI versus 19.9% for angiography-guided PCI, *p* = 0.09). Regarding the precision of stent placement, five side-branch occlusions were observed in the case of patients treated with classic angiography-guided PCI, in contrast to those treated with IVUS-guided PCI when only one side-branch occlusion was reported. However, the study is limited by the small number of adverse events during follow-up and the non-randomized design.

Ali et al. also included patients with advanced CKD (eGFR < 30 mL/min/1.73 m^2^) and chronic coronary syndromes [12], but the authors used a zero-contrast IVUS-guided PCI technique in comparison with the previous study. Patients underwent a low-contrast coronary angiography before the index PCI (at least 1 weak time interval between procedures), with no worsening of kidney function at 21 h after angiography (creatinine = 3.9 mg/dL, IQR 2.9–4.9, eGFR = 18 ± 8 mL/min/1.73 m^2^, and *p* > 0.05). During follow-up (79 days, median), kidney function was maintained to be stable, without observing statistically significant changes (creatinine = 3.7 mg/dL, IQR 3.0–4.5, *p* = 0.69; eGFR = 18 mL/min/1.73 m^2^, IQR 14–22 mL/min/1.73 m^2^, and *p* = 0.70). Moreover, no patient experienced major cardiovascular and renal events during the follow-up period, including RRT, stent thrombosis, revascularization, myocardial infarction, and death, suggesting a zero-contrast IVUS-guided PCI technique could be performed in patients with advanced CKD with a good safety profile. However, the study is limited by the non-randomized design, small number of patients, and by the lack of a comparator, represented by classic coronary angiography-guided PCI.

Patients with chronic kidney disease were also investigated by Sacha et al., but the authors used a slightly different cut-off (eGFR < 45 mL/min/1.73 m^2^) [10]. An important fact is that individuals with hemodialysis (with preserved diuresis, ≥500 mL/day) were also included per protocol. Patients with chronic coronary syndromes were eligible, but the trial also included patients with myocardial infarction in cases when PCI was performed as a staged procedure. Although the authors explored the efficacy and safety of zero-contrast PCI, a small volume of contrast media (3.5–9 mL) was injected at the end of the procedure to exclude distal complications.

Another particularity of this study was the inclusion of patients with complex coronary lesions, including left main stenosis, three-vessel disease, and vein graft lesions. Patients underwent ultra-low coronary angiography (≤15 mL) before the index PCI (median time interval between procedures was 6 days). The kidney function was similar before and after PCI (creatinine mean change 0.1 ± 0.31 mg/dL, *p* = 0.2; eGFR mean change −0.7 ± 10.9 mL/min/1.73 m^2^, and *p* = 0.8). Patients with hemodialysis median urine output did not differ before and after PCI (respectively, 900 mL/day versus 875 mL/day, and *p* = 0.8). Contrast-induced AKI was observed in two patients after classic angiography, but those patients did not experience a similar adverse renal event after zero-contrast PCI, suggesting the procedure’s safety in high-risk patients. Nonetheless, results are limited by the small number of patients, adverse events, and non-randomized and retrospective design.

Kumar et al. [19] reported a case of a complex patient with non-ST-elevation myocardial infarction, advanced CKD, and several previous hemodialysis sessions for acute kidney injury. In addition, the patient had three-vessel disease (SYNTAX-1 score = 35). The authors performed IVUS-guided rota-assisted zero-contrast PCI technique, with good stents expansion, without coronary dissection, and no pericardial effusion. Moreover, kidney function was maintained to be stable, as only a slight rise in serum creatinine was observed (3.6 mg/dL from 3.4 mg/dL), highlighting the importance of the technique even in complex coronary lesions with a good safety profile.

Another two case reports (Patel et al. and Rahim et al.) [13,20] included patients with stage 4 CKD, diabetes mellitus, and complex coronary lesions: Severe lesions of the distal left main trunk, proximal LAD, and mid- RCA in one case, and lesions of the left main trunk (bifurcation), proximal, and mid LAD in the other one. In both situations, the IVUS-guided PCI procedure was safely performed, and kidney function was stable during follow-up, denoting this approach’s safety even in complex coronary lesions.

The single non-randomized study’s quality was appraised using the Newcastle–Ottawa scale, which is not applicable for the rest of the studies included in our systematic review, case-series, and case reports studies (Table S2).

## 4. Discussion

Our endeavor is to conduct a systematic review to include studies evaluating the efficacy and safety of percutaneous coronary interventions using IVUS and minimal- or zero-contrast in chronic kidney disease patients.

The two recent meta-analyses, mentioned by us in the Introductory section, demonstrated the superiority and safety of performing percutaneous interventions using IVUS/FFR versus conventional PCIs (without IVUS), even emphasizing the reduction of cardiovascular mortality. However, the use of a minimum or zero-contrast and the inclusion of patients with chronic kidney disease have not been reported in the IVUS group [14,15].

As part of the SYNTAX II study protocol, instantaneous wave-free ratio (iFR), FFR, and IVUS were used in selected patients for PCI optimization [21]. Notable changes were observed between SYNTAX II and the previous SYNTAX I trial. Given this sophisticated protocol, one-year outcomes were improved compared to the SYNTAX PCI arm I study in terms of major adverse cardiac and cerebrovascular events, definite stent thrombosis, any revascularization, and any myocardial infarction. Surprisingly, the outcomes of optimized PCIs in the SYNTAX II trial were somewhat similar to those of the CABG arm from the SYNTAX I study [22]. On the other hand, when it comes to CKD patients, as shown in the ISCHEMIA-CKD study, an invasive strategy such as PCI did not reduce the risk of adverse events as compared with a conservative strategy [23]. However, in the ISCHEMIA trial, including the ISCHEMIA-CKD study, intravascular coronary imaging such as IVUS or OCT were rarely used.

The next step forward appeared to be Zhang’s trial [24], in which study populations consisted only of CKD patients (in fact, it was a post-hoc analysis from the ULTIMATE trial) [25], and the comparison was between standard and IVUS-guided procedures. Likewise, the idea of low-contrast procedures in CKD was not taken into account. We also intended to perform a meta-analysis; nevertheless, only one study [16] reported a comparator, the others being only descriptive.

The main consistent idea that emerges from our systematic review is that (given all studies reported so far) contrast-free and IVUS-guided PCI procedures in patients with CKD appear to be safe (both in cardiac and renal outcomes, without renal adverse events and higher kidney preservation) with a comparable efficacy to the conventional procedure, even in complex atherosclerotic lesions. This approach seems promising in renal patients who develop coronary syndromes, in the idea that an invasive procedure without the risks induced by media contrast comes with lower chances of further deterioration of renal function, probably with cardiovascular benefits (due to the use of IVUS).

The idea of using IVUS-guided low-contrast coronary interventions can be applied not only to the population with CKD but also to other patients who have risk factors to develop AKI after PCI. Thus, patients having either advanced age, diabetes mellitus, intra-aortic balloon pump use, cardiogenic shock, or a high number of revascularized vessels could potentially benefit from minimum- or zero-contrast IVUS-guided PCI [26].

Noteworthy, we found that no patient included in the mentioned studies showed renal function deterioration and did not need renal replacement therapy after the zero-contrast IVUS-guided percutaneous procedures. Besides, from a cardiovascular point of view, this technique proved to be safe in terms of cardiovascular outcomes.

Currently, what seems to be missing is at least one randomized controlled trial with a high number of advanced CKD participants, comparing zero-contrast IVUS-guided PCIs with conventional angiography and extending the follow-up period. The (positive) results of such a study would constitute the basis for an indication in the European Guidelines (given that at this time, the ‘zero-contrast’ procedure is not taken into account in the management of renal patients requiring PCI). Moreover, given physicians’ well-known reluctance to refer renal patients with coronary syndromes to angiography (‘therapeutic nihilism’), a paradigm shift by not using contrast could very well change physicians’ perceptions and prognosis of these patients.

Although Sakai et al. reported outstanding results a year after the zero-contrast procedure in advanced renal patients, they are still quite reluctant about the ease of positioning the stent without contrast [16], a matter resolved by other operators through ‘dynamic roadmap’ technology [13]. Even if they had no complications, the same authors would talk about the limitations of the IVUS method to promptly detect acute complications, such as coronary perforation, extensive coronary dissections, or slow-flow or no-flow phenomena, technical issues that seem to be ‘critical drawbacks’ for a large-scale implementation of the technique. In addition, Sacha et al. were more optimistic regarding the ‘steep curve’ of the interventional cardiologists learning program, with no conversion to standard PCI in the study group. This enthusiasm is sustained by the good results obtained in the interventions on the complex left main and saphenous vein graft lesions [10].

In a small study published in the European Heart Journal, Ali et al. reported high procedural success and no complications [12]. Even if this trial (also included in our systematic review) reported only 31 patients, its authors are the only ones who propose a standardized protocol in terms of interventional technique (catheter intubation, guide passage, and road map preparation) and post-procedural follow-up (including the risk of coronary perforation management). Their reported strategy could therefore be utilized in experienced centers and selected high-risk patients with advanced CKD.

With this evidence, we believe that using functional assessments such as FFR/iFR and intravascular imaging of IVUS, could play an important role based on a better PCI optimization tool and could also be applicable to CKD patients. Thus, we highlight the combination of functional ischemia and imaging modality assessment to make low contrast procedures work.

Finally, all of the studies described above mention the need for a randomized control trial to lower the selection bias and raise the level of evidence. However, Sakai et al. found it impossible to draw up a randomized study protocol due to ‘ethically challenging’ reasons in this very high-risk group [16]. Acknowledging most operators’ substantial experience and the safety of contemporary IVUS and FFR procedures, we believe that just a small step is missing for zero-contrast PCI to receive an indication in the guidelines. In this regard, a good example is the left atrial appendage occluders indication in the ESC Atrial Fibrillation Management Guidelines (with an IIb class indication and level of evidence B, and a net higher risk of complications than contrast-free PCI).

## 5. Conclusions

Beyond its burden and setbacks, chronic kidney disease carries the heavy load of a higher rate of cardiovascular complications, of which coronary syndromes hold the leading place. Conventional percutaneous angiographic procedures are there to save lives, although paradoxically, they may negatively impact CKD patients’ mortality due to frequent contrast-induced acute kidney injuries. These complex and bidirectional consequences might soon be effectively hampered by safer low-to-zero contrast IVUS-guided PCI procedures after mandatory and rigorous evidence-based validation.

## Figures and Tables

**Table 1:** Inclusion criteria for selected studies according to the PICOS statement.

Criteria	
Population	Patients (aged > 18 years) with chronic kidney disease and acute or chronic coronary syndromes.
Intervention	Minimum-contrast or zero-contrast intravascular ultrasound guided percutaneous coronary interventions.
Comparators	Angiography guided percutaneous coronary interventions.
	None.
Outcomes	Efficacy and/or safety
Type of Study	Randomized or non-randomized studies, observational studies, case series, and case reports.
Language	English.

**Table 2:** General characteristics of studies included in systematic review.

Study, Year	Design	Patients, No	Age (Years), Median/Mean	Setting	Intervention	Comparator	Outcomes	Follow-Up
Ali et al., 2016 [12]	Retrospective analysis (case series), single center	31	66 ± 11	Patients with advanced CKD (stages 4–5) and stable CAD	Zero-contrast IVUS guided PCI	N/A	-Requirement of RRT -Stent thrombosis -Revascularization -Myocardial infarction -Death	79 days (median)
Sakai et al., 2018 [16]	Non-randomized, multicenter	184	74 ± 7 (angiography-guided PCI) 76 ± 9 (IVUS-guided PCI)	Patients with CAD, elective PCI, and CKD stages 4–5 (excluding hemodialysis)	IVUS-guided minimum-contrast PCI (98 patients)	Angiography-guided PCI (86 patients)	-All-cause mortality -Cardiac death -Non-cardiac death -Requirement of RRT	12 months
Sacha et al., 2019 [10]	Retrospective analysis, single center	20	73.7 ± 12.8	Patients with CKD (eGFR < 45 mL/min/1.73 m^2^) including hemodialysis (preserved urine output) admitted due to acute coronary syndrome or in elective setting	Zero-contrast IVUS guided PCI	N/A	During hospitalization: -Change in creatinine/eGFR -Acute kidney injury -Requirement of RRT (in patients without dialysis) -Periprocedural myocardial infarction -Distal embolization During follow-up: Acute coronary syndrome Stent thrombosis Repeat revascularization StrokeRequirement of RRT (in patients without dialysis) Death	3.2 months (median)
Kumar et al., 2020 [19]	Case report	1	54	CKD patient with recent history of few cycles of hemodialysis for acute on chronic kidney disease	IVUS-guided rota-assisted left main zero-contrast PCI	N/A	During hospitalization: -Post stenting pericardial effusion -Post intervention symptoms -Hemodynamic instability -Change in creatinine	-
Patel et al., 2020 [20]	Case report	1	70	CKD stage 4 and history of hypertension, type 2 diabetes mellitus	IVUS-guided PCI of RCA with zero-contrast, and PCI of distal LM to LAD using minimum-contrast	N/A	During hospitalization: -Changes in renal function -Post intervention symptoms During follow-up: -Changes in renal function -Post intervention symptoms	1 week
Rahim et al., 2019 [13]	Case report	1	57	CKD stage 4 and a history of HIV, diabetes mellitus	Zero-contrast PCI of LM (bifurcation),LAD with LV support	N/A	During hospitalization: -Procedural harm -Procedural success	6 months

CAD—coronary artery disease; CKD—chronic kidney disease; eGFR—estimated glomerular filtration rate; IVUS—intravascular ultrasound; LAD—left anterior descending artery; LM—left main trunk; LV—left ventricle; PCI—percutaneous coronary intervention; RCA—right coronary artery; RRT—renal replacement therapy.

**Table 3:** Results reported in studies included in the systematic review.

Author, year	Outcomes	Results	
Sakai et al., 2018 [16]	IVUS-guided PCI versus angiography-guided PCI		
	All-cause death	6 (6.4%) vs. 6 (7.8%) patients	*p* = 0.85
	Cardiac death	2 (2.2%) vs. 2 (2.6%) patients	*p* = 0.98
	Non-cardiac death	4 (4.4%) vs. 4 (5.3%) patients	*p* = 0.79
	Requirement of RRT	3 (3.2%) vs. 11 (13.6%) patients	*p* = 0.01
Ali et al., 2016 [12]	Stent thrombosis	0 (0%) patients	
	Revascularization	0 (0%) patients	
	Myocardial infarction	0 (0%) patients	
	Death	0 (0%) patients	
	Requirement of RRT	0 (0%) patients	
Sacha et al., 2019 [10]	During hospitalization		
	Change in creatinine (mg/dL)	0.1 ± 0.31	*p* = 0.2
	Change in eGFR (mL/min/1.73 m^2^)	−0.7 ± 10.9	*p* = 0.8
	AKI after zero-contrast PCI	2 (10%) patients	
	Requirement of RRT	0 (0%) patients	
	Periprocedural myocardial infarction	1 (5%) patient	
	Distal embolization	1 (5%) patient	
	During follow-up		
	Acute coronary syndrome	0 (0%) patients	
	Stent thrombosis	0 (0%) patients	
	Repeat revascularization	0 (0%) patients	
	Stroke	0 (0%) patients	
	Requirement of RRT	0 (0%) patients	
	Death	1 (5%) patient	
Kumar et al., 2020 [19]	During hospitalization		
	Post stenting pericardial effusion	0	
	Post intervention symptoms	0	
	Hemodynamic instability	0	
	Change in creatinine	0.2 mg/dL	
Patel et al., 2020 [20]	During hospitalization		
	Changes in renal function	0	
	Post intervention symptoms	0	
	During follow-up		
	Changes in renal function	0	
	Post intervention symptoms	0	
Rahim et al., 2019 [13]	During hospitalization		
	Procedural harm	0	
	Procedural success	0	

AKI—acute kidney injury; eGFR—estimated glomerular filtration rate; IVUS—intravascular ultrasound; PCI—percutaneous coronary intervention; RRT—renal replacement therapy.

## Data Availability

The data presented in this study are available on request from the corresponding author.

## Supplementary Materials

The following are available online at https://www.mdpi.com/article/10.3390/jcm10091996/s1, Table S1: Preferred Reporting Items for Systematic Review and Meta-Analysis (PRISMA) checklist, Table S2: Quality assessment using Newcastle–Ottawa scale.
