# Supplementary material for: Safety and Efficacy of Minimum- or Zero-Contrast IVUS–Guided Percutaneous Coronary Interventions in Chronic Kidney Disease Patients: A Systematic Review
Source: J Clin Med. 2021 May 6;10(9):1996. doi: 10.3390/jcm10091996 (PMC8125490; doi:10.3390/jcm10091996)
Supplement: Supplementary file 1 [file jcm-10-01996-s001.zip › Table S2.pdf]

Table S4. Quality assessment using Newcastle-Ottawa scale

	**Sakai et al, 2018**
Selection:	
Representativeness of the exposed cohort	*
Selection of the non-exposed cohort	*
Ascertainment of exposure	*
Demonstration that outcome of interest was not present at start of study	*
Comparability	
Comparability of cohorts on the basis of the design or analysis controlled for confounders	
Outcome	
Assessment of outcome	*
Was follow-up long enough for outcomes to occur?	*
Adequacy of follow-up	
**Total**	6 stars

Good quality: 3 or 4 stars in selection domain AND 1 or 2 stars in comparability domain AND 2 or 3 stars in outcome/exposure domain. **Fair quality:** 2 stars in selection domain AND 1 or 2 stars in comparability domain AND 2 or 3 stars in outcome/exposure domain. **Poor quality:** 0 or 1 star in selection domain OR 0 stars in comparability domain OR 0 or 1 stars in outcome/exposure domain.
